# Role of female community health volunteers in ischemic stroke prevention, identification, referral and rehabilitation in Nepal

**DOI:** 10.1016/j.amsu.2021.102893

**Published:** 2021-10-08

**Authors:** Babin Basnet, Jayant Kumar Yadav, Bikram Prasad Gajurel, Yow Ka Shing, Bipin Kandel, Gaurav Nepal

**Affiliations:** Department of Internal Medicine, Tribhuvan University Teaching Hospital, Kathmandu, Nepal; Department of Neurology, Tribhuvan University Teaching Hospital, Kathmandu, Nepal; Department of Internal Medicine, Tribhuvan University Teaching Hospital, Kathmandu, Nepal; Rani Primary Health Care Center, Biratnagar 56613, Morang, Nepal

**Keywords:** FCHV, Female community health volunteers, Stroke, Ischemic stroke, Nepal

## Abstract

For the past three decades, female community health volunteers (FCHVs) have been at the forefront of Nepal's health map and have contributed significantly to its improving health indicators such as maternal mortality rate and infant mortality rate. Given the changing epidemiology of Nepal and the shift of burden from communicable to non-communicable diseases (NCDs), it is important to revitalize their role with the changing times. The prevalence of ischemic stroke in Nepal is on the rise. However, very few people make it to the hospital within the time frame for thrombolysis and the patient's knowledge about ischemic stroke seems to play a major part. FCHVs can play a significant role in improving ischemic stroke care by raising awareness about the condition, its risk factors, and informing the public about the need for timely treatment. They can help screen for common risk factors such as obesity, hypertension and diabetes as well as monitor for treatment in previously diagnosed individuals. Randomized controlled trials have shown to yield favorable results in NCDs with engagement of FCHVs. With proper training and support, they can play an important role in improving ischemic stroke care in low- and middle-income countries like Nepal.

Stroke is known to be the second leading cause of death globally and the third leading cause of premature death and disability as measured in Disability Adjusted Life Years (DALY) as shown by the Global Burden of Disease Study. Developing countries share the major burden of stroke comprising 75% of deaths from stroke and 81% of stroke-related DALYs [1]. According to data of the largest tertiary referral hospital of Nepal, ischemic stroke accounted for 36.5% of all admitted cases in neurology ward and 73.8% of all admitted cases of stroke [2]. Despite the high prevalence of stroke, a recent study from Nepal showed that only 20% of patients suffering from ischemic stroke made it to the hospital within 4.5 h time frame for intravenous thrombolysis. Factors that led to the early arrival to hospital included awareness of stroke symptoms, education level and local accessibility to hospital care [3]. Hence, educating community about signs and symptoms, risk factors, possible treatment can have tremendous impact in improving ischemic stroke care.

In the 1980s, Nepal started a programme of Female Community Health Volunteers (FCHVs), commonly known as “mahila swoyemsewika”, which means “female volunteer. Since its inception, their roles were to support family planning, maternal and child health services. Gradually, their roles were expanded to include other programmes [4]. FCHVs are an integral part of community-based health programs and act as bridge between families and communities to tertiary health systems. There is at least one FCHV serving in each ward of the village development committee, the smallest local administrative body, in Nepal. They are local women who are selected by mothers’ group and work voluntarily in the community. After being selected, they receive 18 days of training on maternal health, newborn care, family planning, and nutritional counselling [5]. At the initial phase of the programme, these volunteers are assigned to promote and distribute the family planning commodities. Over time, they assumed an important role in health promotion including implementation of maternal child health programs as well as treatment and referral for common conditions such as childhood pneumonia and diarrhea [6,7]. Their role has been instrumental in achieving a significant decline in maternal mortality rate, infant mortality rate and millennium development goals in Nepal [8].

Given the changing epidemiology of Nepal and the shift of burden from communicable to non-communicable diseases (NCDs), the need to revitalize the role of FCHVs has been felt [9]. Given the proximity of FCHVs with community people and trust, they can play an active role in health awareness, educating the public on the signs and symptoms of stroke and the importance of primary prevention, including the benefits of healthy dietary habits, exercise, and cessation of smoking and alcohol [4]. In rural Nepal, misconceptions prevail; many in such communities believe that neurological conditions such as stroke are God's punishment to an individual and seek faith healers before physicians, delaying the urgent care they need [10]. FCHVs may assist in demystifying such myths through their presence as community health advocates and educators.

As much as it is important for the community to recognize stroke, timely transfer to hospital for treatment is equally vital. The time window for thrombolysis in ischemic stroke is 4.5 h [11]. In Nepal, neurologists, CT and MRI services are largely concentrated in major cities [10]. Once early recognition is made, early referral and transfer to a tertiary center is critical to maximize a patient's window of opportunity for thrombolysis. FCHVs have the potential to facilitate this process and can play a pivotal role in increasing the community's accessibility to tertiary acute stroke care.

With further training, FCHVs can be taught to evaluate for common risk factors of ischemic stroke, such as Body Mass Index (BMI) measurement, screening for hypertension through blood pressure measurement, and monitoring of diabetes control through home blood glucose monitoring [12]. This can be useful in two fronts – diagnosing cardiovascular comorbidities early and monitoring control of known conditions. Those with abnormal screening tests or poor control of underlying conditions can be referred for further evaluation and early treatment. This can be initiated and followed through by the FCHV through appropriate counselling and assistance in scheduling of follow-up visits. In the long run, FCHVs potentially contribute to a valuable longitudinal health evaluation and management for patients in the community.

Existing FCHV programmes in Nepal have proved to be highly successful in primary and secondary prevention. In a randomized controlled trial (RCT) by Neupane et al., 1638 community participants were recruited, with FCHVs providing home visits every 4 months for lifestyle counselling and blood pressure monitoring in the intervention group [13]. The mean systolic blood pressure at one year was significantly lower in the intervention group than in the control group for all cohorts: the difference was −2·28 mm Hg (95% CI −3·77 to −0·79, p = 0·003) for participants who were normotensive, −3·08 mm Hg (−5·58 to −0·59, p = 0·015) for participants who were prehypertensive, and −4·90 mm Hg (−7·78 to −2·00, p = 0·001) for participants who were hypertensive. [13]. Neupane's study was otherwise known as the Community-based management of hypertension in Nepal study (COBIN) trial which underwent subsequent retrospective cost effectiveness and budget impact analysis, which showed the programme to be highly cost-effective with a first-year budget impact of between US$7·1 to US $10·8 million [14].

In the Community-based intervention for management of diabetes in Nepal (COBIN-D) trial, a cluster RCT involving 244 adults with type 2 diabetes mellitus by Gyawali et al., FCHV-delivered intervention via education, screening and counselling was shown to be associated with a statistically significant greater reduction in mean fasting blood glucose [12]. In their programme, 20 FCHVs were selected and trained for five days on the overview of diabetes, its modifiable and non-modifiable risk factors, screening blood glucose level, blood pressure, height, weight, home based health education and counselling, and effective communication for behavior change. They then provided home visits once every 4 months for health promotion counselling and blood glucose monitoring. At baseline, the mean (SD) fasting blood glucose level was 156.06 (44.48) mg/dL (158.48 [45.50] mg/dL in the intervention group and 153.43 [43.39] mg/dL in the control group). At the 12-month follow-up, the mean fasting blood glucose decreased by 22.86 mg/dL in the intervention group, whereas it increased by 7.38 mg/dL in the control group. The mean reduction was 27.90 mg/dL greater with the intervention (95% CI, −37.62 to −18.18 mg/dL; P < .001). In the study's secondary outcome analyses, there was a greater decline in mean systolic blood pressure in the intervention group than in the control group (−5.40 mm Hg; 95% CI, −8.88 to −1.92 mm Hg; P = .002), and a significant difference in the intake of oral hypoglycemic medication between the two groups (relative risk, 1.35; 95% CI, 1.1 to 1.74; P = .02) [12].

FCHVs are volunteers, meaning they are not paid for their services, and there are no direct benefits to the job apart from elevated social standing in the community for their work [16]. However, a recent municipality cross-sectional survey showed that 99% of FCHVs were willing to contribute to areas of health promotion and screening activities without any financial incentives [17]. Many of them are intrinsically and altruistically driven, and the interest in primary and secondary prevention suggests the community is increasingly cognizant of the importance of addressing non-communicable disease, which is seeing a precipitous rise in the current Nepal health climate [[9], [15]].

Despite the intrinsic interest of FCHVs toward public health promotion, there are challenges providing this on a practical level. Firstly, FCHV literacy levels throughout the country are not uniform. Given the complex nature of stroke presentation and prevention, it may be difficult to achieve consistent standards in the community health role played by FCHVs in this area. Secondly, adequate government and local organizational support is required to plan and provide effective training for FCHVs prior to the programme, which has been shown by previous RCTs to be crucial in ensuring quality and effectiveness of FCHV-delivered service. This will not be easy to standardize and execute on a nation-wide level given the subtle differences in the workings of each municipality in Nepal. Thirdly, there are potential barriers in the public's existing beliefs in health and healthcare, as well as the trust placed in FCHVs. Sarita Panday's qualitative interviews and focused group discussions with FCHVs showed that many of them faced issues of lack of trust of the public in the volunteers, traditional beliefs (such as seeking healthcare from faith healers) and perceived indignities experienced when using health centers [18]. These perceptions can only be adequately addressed through awareness, education and continued sociopolitical support of the FCHV volunteer groups, whilst being sensitive to the unique sociocultural aspects specific to each region in Nepal.

To address these challenges, it is imperative for an FCHV-led ischemic stroke prevention and support programme to be bolstered by appropriate government support and resources. Robust education programmes, including workshops, counselling role-playing sessions, and home visit stimulations are recommended to increase the effectiveness of the healthcare volunteers. These must be tailored to address specific barriers and challenges that are unique to the local population, as previous qualitative studies have alluded to. Links to tertiary healthcare providers are crucial in ensuring there is an established community-to-hospital referral system for professional assistance, be it in the context of acute stroke or chronic disease management. During the programme, a two-way dynamic should exist, as the input from the healthcare system of the individual and the reverse are equally important in upkeep of longitudinal healthcare of the community. In this aspect, FCHVs’ commitment and time spent connecting with locals on the ground is critical in providing pertinent two-way feedback for the system to improve in the future. Throughout the programme, it should be borne in mind that FCHVs are involved on a purely voluntary basis, and they should be continually supported via appropriate incentives and reward systems, especially for long-term volunteers who have made significant contributions.

## Conclusion

Ischemic stroke has a huge economic and health burden on patients, family members and the nation as a whole. FCHVs are members of the community who share the same living region, language and culture of the residents. Equipped with appropriate knowledge and skillsets, FCHVs potentially form a pivotal contact point between the community and the healthcare system, and have the capacity to become effective health advocates for their beneficiaries. Their contribution can make a significant difference in the prevention, early identification, referral and rehabilitation of ischemic stroke in limited-resource country like Nepal.

## Sources of funding

Not funded.

## Consent

Not applicable.

## Ethical approval

Not applicable.

## Author contribution

GN, JKY and BPG conceived the study. BB, GN and JKY performed literature search, interpretation the available literatures and drafted the manuscript. YKS, BK, BPG and GN critically revised the manuscript for intellectual content. All authors read and approved the final manuscript. GN and BPG are guarantors of the paper.

## Declaration of competing interest

None.
